# 

**Published:** 1868-08

**Authors:** 


### INDEX.

### A.

Abstract of Proceedings of the Buffalo Medical Association, 81, 132, 167, 212, 256, 300, 336, 370, 455.
Abuse of Athletic Games ................. 227
Action of Iodide of Potassium increased by Ammonia ..................... 185
Acupressure and its Effects .............. 106
Administration of Drugs ................. 178
Addresses ............................... 238
Address to the Graduates of Medical Department of the University of Buffalo. By Prof. Thos. F. Rochester, M. D ... 297
Admitted to the U. S. Navy .............. 363
Advantages of Combination in Therapeutics. By D. E. Chace, M. D .......... 405
Aged Primipara .......................... 36
Amputation of the Cervix Uteri. By J. F. Miner, M. D ......................... 136
American Opium from Vermont, Notes on, 157
American Medical Association, 323, 324, 373, 423.
Aneurism of the Femoral and Lower Portion of the Iliac Arteries, Ligation of the External Iliac. By J. F. Miner, M. D .............................. 249
Annual Meeting of the Erie County Medical Society ............................ 200
Annual Commencement Exercises in the Buffalo Medical College—List of Graduates .............................. 276
Announcement of the Starling Medical College ............................. 444
Appetite for Medicines .................. 111
Arsenic in Skin Diseases ................ 316
Ascarides, Treatment of .................. 36
Atrophine as an Antidote to Opium ...... 173
Atlantic Monthly and Dr. Henry J. Bowditch ............................... 239

### B.

Beer, Nutriment of ...................... 110
Belladonna as an Antidote to Opium. By O. C. Gibbs, M. D .................... 258
Biographical Sketch of the Late Dr. John O'Reilly ............................. 251
Bishop Coxe on Science .................. 347
Black Snow .............................. 35
Bleeding in Pneumonia, Rapid Recovery .. 231
Books Received, 40, 78, 119, 158, 238, 283, 322, 363, 404, 482.
Boys Who Smoke ......................... 179
Braman, Dr., U. S. A., Murder of ......... 62
Brain and Mind Action ................... 240
Bromides in Cases of Lead or Mercurial Poisoning ............................ 229
Bromide of Potassium .................... 404
Bromide of Potassium in Epilepsy ........ 476
Buffalo Medical Association, Abstract of Proceedings, 81, 132, 167, 212, 256, 300, 336 370.

### C.

Case of Injury to the Median Nerve. By J. R. Lothrop, M. D ..................... 10
Causes of Congenital Club-foot, with Remarks upon the Time for Operation. By J. F. Miner, M. D .................. 41
Critical Review—Tuberculosis and Pulmonary Phthisis .......................50, 97
Carbolic Acid Solutions, Strength of ...... 110
Carbolic Acid in Cutaneous Disease ....... 198
Carbolic Acid in the Treatment of Boils, Whitlows and Abscesses ............. 179
Carbolic Acid as a Remedial Agent ....... 216
Carbolic Acid in Scarlatina ................ 267
Carbolic Acid ............................ 387
Caries of the Humerus. By J. F. Miner, M. D ................................. 209
Cataract, Operation for Extraction of ..... 163
Case of Capsular Secondary Cataract. By Eugene Smith, M. D. ................ 459
Case of Congenital Deformity of the Hands, 170
Case of Incised Wound of the Abdomen, with Transverse Division of the Mesenteric Artery ........................ 182
Case Followed by Tetanus Amputation—Death upon the Sixth Day. By T. Mack, M. D .......................... 201
Carpenter, Paul D., M. D. Case of Inflammation of the Stomach ............... 139
Case of Polypus Arising from the Urethra of a Female; Complicated, with four Bands of Adhesion of the Vagina, and complete acclusion of the Os and Cervix Uteri. By W. H. Williams, M. D., 325
Cervix Uteri. Amputation of. By J. F. Miner, M. D ........................ 136
Cholera Fungus .......................... 112
Chloroform, New Method of Administering .................................. 199
Chloroform, Death from .................. 315
Chronic Catarrh, Pharyngitis, Laryngitis, Bronchitis, etc. By Wm. M. Cornell, M. D ............................... 327
Chace, D. E., M. D. Advantage of Combination in Therapeutics ............... 405
Chemistry of the Cinchona Bark (Cincho Quinine) .............................. 432
Cincho Quinine .......................... 444
Clark, E. A., M. D. Method of Treating Fracture of the Olecranon Process and Head of the Humerus ................. 30
Clinical Remarks upon Surgical Cases in the Buffalo General Hospital—Amputation of the Cervix Uteri. By J. F. Miner, M. D ........................ 136
Clinical Remarks upon Surgical Cases in the Buffalo General Hospital. By J. F. Miner, M. D ...................... 163
Clinical Remarks upon Surgical Cases in the Buffalo General Hospital. By J. F. Miner, M. D ...................... 209

Clinical Remarks upon Surgical Cases in the Buffalo General Hospital—Aneurism of the Femoral and Lower Portion of the Iliac Arteries—Ligation of the External Iliac. By J. F. Miner, M. D., 247
Clinical Remarks upon Surgical Cases in the Buffalo General Hospital—Congenital Cataract, Perineal Section for the Relief of Impassable Stricture. By J. F. Miner, M. D. .......... 333
Clinical Remarks upon Surgical Cases in the Buffalo General Hospital—Hydrocele—Operation for Occlusion of Varicose Veins—Tumor from the Carotid Region—Tumor from the Axilary Space. By J. F. Miner, M. D. .......... 365
Cornell, Wm. M., M. D., L. L. D. Dysentery Treated by Nux Vomica .......... 28
Cornell, Wm. M., L. L. D. Chronic Catarrh, Pharyngitis, Laryngitis, Bronchitis, etc. .......... 327
Combination of Chloroform with Opiates to Relieve Pain .......... 460
Compound Dislocation of the Elbow-joint —Case Followed by Tetanus Amputation—Death upon the Sixth Day. By T. Mack, M. D .......... 201
Correction of Misplaced Cuts .......... 444
Correction of Names .......... 282
Commencement Exercises in the Buffalo Medical College—List of Graduates... 276
Coxe, Bishop, Pastoral Letter by .......... 275
Coxe, Bishop, on Science .......... 347
Communicability of Phthisis .......... 316
Concerning the Obstetrical Properties of Ergot of Rye .......... 398
Contagion .......... 223
Croup Treated by Sulphur .......... 187
Creasote in Typhoid Fever .......... 436
Cure for Headache .......... 188

### D.

Dangers of Opium in Mania a Potu. By J. O. Whitney, M. D .......... 46
Davis, Prof. N. S. Vaginimus .......... 105
Dead Alive .......... 109
Defect, Monstrosity by .......... 111
Death from Hypodermic Injection .......... 194
Death by Careless Renewal of Prescription, 240
Death from Chloroform .......... 315
Death of Dr. Arnold .......... 324
Death of Dr. Alden March .......... 442
Death from Pyaemic Poisoning .......... 404
Deformity of Hands—Case of Congenital, 170
Dean, H. W., M. D. Statistics from one hundred patients, whose First and Second Confinements occurred under my observation, designed to exhibit the comparative duration of first and second labors .......... 206
Diarrhœa of Infants, Use of Pepsine in. By Jas. S. Hawley, M. D .......... 1
Diphtheritic Membrane, Solubility of..... 180
Disease, Treatment of Malarial .......... 183
Dislocation, Compound, of the Elbow-joint —Case Followed by Tetanus Amputation—Death upon the Sixth Day. By T. Mack, M. D .......... 201
Drugs, the Administration of .......... 178
Dysentery, Treated by Nux Vomica. By Wm. Cornell, M. D., L. L. D .......... 28

### E.

Eastman, Prof. Sandford, M. D. Introductory Lecture to the Students of the Medical Department University of Buffalo .......... 121
Effects of Noticing Quacks .......... 60
Embalming, or Nekrosoziac .......... 74
Emmett, Dr., on Vesico Vaginal Fistula... 80
Empiric, the .......... 271
Encephaloid Tumor weighing 36⅜ pounds, in a child eight and a half years of age, Reported by C. C. F. Gay, M. D .......... 161
Erie County Medical Society, Meeting of, 161, 239, 200.
Ergot of Rye, Concerning the Obstetrical Properties of .......... 398
Eve, Prof. Paul F. Contribution on the History of Hip-joint operations. Rejoinder to a Review of .......... 21
Eve, Prof. Paul F. Reply to Dr. Otis' Rejoinder .......... 91
Every Saturday .......... 200
Examination of Dr. Chas. P. Powers..... 80
Excursion to New Orleans .......... 284
Eye, Recent Improvement in Operative Surgery of the .......... 268

### F.

Fare to the Meeting of the American Med-Association, New Orleans .......... 364
Fistula, Vesico Vaginal, Dr. Emmett on.. 80
Flint, Prof., Invited to a Public Dinner... 37
Fluid Extracts .......... 350
Fracture of the Olecrenon Process, and Head of the Humerus, Method of Treating. By E. A. Clark, M. D ...... 30
Fungi and Disease .......... 112
Fungus, Cholera .......... 112

### G.

Gay, C. C. F., M. D. Phymosis with Impermeable MeatusUrinarius,Operation 18
Gay, C. C. F., M. D. Intestinal Invagination, Strangulation, Sloughing and Discharge Per Rectum of Fourteen inches of Intestine, with fatal Hemorrhage.. 18
Gay, C. C. F., M. D. Retroversion of the Impregnated Uterus, and Spontaneous Reposition .......... 87
Gay, C. C. F., M. D. Encephaloid Tumor weighing 36⅜ lbs. in a Child eight and a half years of age—Death, Autopsy. Reported by .......... 163
Gay,C.C.F.,M.D. Hernia, with Remarks, 297
Gibbs, O. C., M. D. Strangulated Hernia, 208
Gibbs, O. C., M. D. Belladonna as an Antidote to Opium .......... 258
Gibbs, O. C., M. D. Venesection as a Means for the Arrest of Unavoidable Hemorrhage in Placenta Praevia ..... 310
Genuine Hermaphrodite .......... 184
Gross, Prof. S. D., Letter from—American *vs.* European Medical Science .......... 437
Guy's Hospital, Pneumonia, Bleeding, Rapid Recovery .......... 231
Gynæcological Society of Boston ......284, 471

### H.

Hawley, Jas. S., M. D. Use of Pepsine in Diarrhœa of Infants .......... 1
Hermaphrodite—A Genuine .......... 184
Hernia, Strangulated. By O. C. Gibbs, M. D .......... 208
Hernia, with Remarks. By C. C. F. Gay, M. D .......... 297
Hip-joint Operations—Rejoinder to a Reply to a Review of Prof. Eve's Contribution on the History of .......... 21
Hip-joint Amputation, Result of .......... 38
Homœopathy, Modern .......... 63
Hypodermic Injection of Remedies ....... 192
Hypodermic Injection, Death from ....... 194

Hygiene—An Address to the Graduates of the Medical Department of the University of Buffalo. By Prof. Thos. F. Rochester, M. D. 385
Hypodermic Method of Injection 354

I.

Immoral Publications 160
Injury to the Median Nerve, A case of. By J. R. Lothrop, M. D. 10
Intestinal Vagination, Strangulation, Sloughing, and Discharge of fourteen inches of Intestines—Hemorrhage. By C. C. F. Gay, M. D 18
Introductory Lecture to the Students of the Medical Department, University of Buffalo. By Prof. Sandford Eastman, M. D 121
Inflammation of the Stomach, A case of. By Paul D. Carpenter, M. D 139
Internal Inflammation, Modern Treatment of Acute 146
Indiana in want of a Medical Journal 155
Injection, Hypodermic, Death from 194
Inoculability of Phthisis 353
Inverted Uterus Reduced after four and a half Years' Duration 364

J.

Journal of Insanity 404

L.

Laceration of the Spleen in Pregnancy 141
Law Regulating the Practice of Medicine in Ohio 145
Law Regulating the Practice of Medicine and Surgery in Pennsylvania 400
Late in our November number 159
Lake Erie Medical Society Abstracts of Proceedings 413
Lee, Prof. Charles, Letter from 48
Lee, Prof. Charles, in Nashville, Tenn 38
Legal Regulation of Medical Practice 399
Ligation of the External Iliac Artery. By J. F. Miner, M. D 249
Life, Prof. Owens' Conclusions on the Origin of Species and Nature of 261
Lothrop, J. R., M. D., A case of Injury to the Median Nerve 10

M.

Mania a Potu, Dangers of Opium in. By J. O. Whitney, M. D. 46
Method of treating Fracture of the Olecranun. Process and Head of the Humerus. By E. A. Clark, M. D 30
Medicine, Value of 66
Medical Association, Abstract of Buffalo, 81, 132, 167, 212, 256, 300, 336, 370, 455.
Medicine Received 120, 159
Medical Progress, Sir James Simpson on 171
Medical Papers presented at the Annual Meeting of the Medical Society of the State of New York 273
Meeting of the American Medical Association 359
Meeting and Organization of Medical Journalists 401
Medical Practice, Legal Regulation of 399
Meeting of the State Commissioners to Locate an Insane Asylum in the Eighth Judicial District 478
Meeting of the Erie County Medical Society 444
Medical Volcanic Eruption—American *vs.* European Science—Letter from Prof. S. D. Gross 437
Miner, J. F., M. D., Causes of Congenital Club Foot, with Remarks upon the time for Operation 41
Miner, J. F., M. D., Clinical Remarks upon Surgical Cases in the Buffalo General Hospital. Amputation of the Cervix Uteri 136
Miner, J. F., M. D., Clinical Remarks upon Surgical cases in the Buffalo General Hospital. Operation for Extraction of Cataract 163
Miner, J. F., M. D., Clinical Remarks upon Surgical Cases in the Buffalo General Hospital—Caries of the Humerus—Artificial Pupil—Glandular Tumor Removed from the Superior Carotid Triangle 209
Miner, J. F., M. D.. Clinical Remarks upon Surgical cases in the Buffalo General Hospital—Aneurism of the Femoral and lower portion of the Iliac Arteries—Litigation of the External Iliac 249
Miner, J. F., M. D., Clinical Remarks upon Surgical cases in the Buffalo General Hospital—Congenital Cataract—Perineal Section for Relief of Impassable Stricture 333
Miner, J. F., M. D., Clinical Remarks upon Surgical cases in the Buffalo General Hospital—Hydrocele—Operation for Occlusion of Varicose Veins—Tumor from the Carotid Region—Tumor from Axilary space 365
Miner, J. F., M. D., Remarks upon different modes of treating the Pedicle in Ovariotomy—Suggestion of a new method, with Report of an Illustrative case 418
Monstrosity by Defect 111
Morbus Coxarius 230
Monstrosity of Sex 393
Monstrosity, Singular 190

N.

Nerve, Median. A case of Injury to. By J. R. Lothrop, M. D 10
Nekrosoziac, or Embalming 74
News Items 120
New Treatment of Acute Rheumatism 175
New Method of Administering Chloroform 199
New Medical Journal 444
Noticing Quacks, Effects of 60
Notes on American Opium from Vermont 157
Nutriment of Beer 110
Nux Vomica, Dysentery Treated by. By Wm. M. Cornell, M. D., L. L. D 28

O.

Obituary. Death of Geo. Jay Sweet, M. D. 79
Death of Dr. Arnold 324
Death of Dr. Alden March 442
Death of Dr. J. R. Lothrop 479
Death of Dr. Charles D. Meigs 482
Ohio Law Regulating the Practice of Medicine 145
On the use of Bromides in cases of Lead or Mercurial Poisoning 229
Opium. Notes on American Opium from Vermont 157
Opium Poisoning, Atropine as an Antidote to 173
Opium, Belladonna an Antidote to 258
Opening of the Woman's Medical College, New York 191
Opening for a Physician 284
O'Reilly, Late Dr. John. Biographical Sketch of 250

Otis, Dr., Rejoinders to Dr. Eve's Reply.. 91
Our Journal .......................... 200
Owen's, Prof., Conclusions on the Origin of Species, and Nature of Life ........ 261
Oxygen, Carbon, Nitrogen, Hydrogen, Phosphorus and Sulphur changed by Adam's Sin .......................... 324
Ozone .................................. 73

P.

Poisoning, Use of Bromides in cases of Lead or Mercurial.................... 229
Pastoral Letter. By Bishop Coxe ........ 275
Papers Presented at the Annual Meeting of the Medical Society of the State of New York .......................... 273
Paralysis, Remarkable case of, from Injury .............................. 341
Pepsine, Use of, in Diarrhœa of Infants. By Jas. S. Hawley, M. D.............. 1
Powers, Chas. P., Examination of........ 80
Phthisis, Communicability of............ 316
Phthisis, Inoculability of................ 353
Polypus, Arising from the Urethra. By Wm. H. Williams, M. D.............. 325
Primipara, an Aged .................... 36
Profession. Room in. Letter and Answer 75
Progressive Locomotor Ataxia, or Duchenne's Disease ......................... 461
Pregnancy, Laceration of the Spleen in ... 141
Puerperal Eclampsia. By C. C. F. Gay, M. D.............................. 445
Pulmonary Phthisis and Tuberculosis. A Critical Review....................50, 97
Pyromania. Paper read before the Erie County Medical Society. By Wm. C. Phelps, M. D......................... 241

Q.

Quacks, Effects of Noticing .............. 60
Quacks and Quackery.................... 264

R.

Result of Hip-joint Amputations ......... 38
Review, Critical. Tuberculous and Pulmonary Phthisis.....................50, 97
Remarks on Books Received ............. 80
Remarkable Vitality of New-born Child.. 145
Remedy for Tooth-Ache.................. 172
Remedial Agent, Carbolic Acid as ........ 216
Renewal of Prescription, Death by Careless .................................. 240
Recent Improvement in Operative Surgery, 268
Resolutions upon Examination for Life Insurance.......................... 265
Remarkable Case of Paralysis from Injury, 241
Reviews—Addison, Writings of ......... .. 280
Aitkins' Science and Practice of Medicine. Second Edition... ............ 115
Aitkins' Science and Practice of Medicine................................ 198
Anatomy and Histology of the Human Eye ................................. 76
Babcock. Notice of Set of Instruments, 281
Badenhamer. Practical Observations on Etiology, Pathology, Diagnosis and Treatment of Anal Fissure........... 281
Bozeman. Operation of Vesico Vaginal Fistula without the aid of assistance.. 320
Bumstead. Atlas of Venereal Diseases. 157
Butler's Physician's Diary............. 236
Byford. Fibrous Tumor Removed from Anterior Walls of the Uterus......... 321
Catalogue of Surgical Instruments Manufactured by W. F. Ford ............ 322
Capron. Annual Report of the Commissioners of Agriculture................ 234
Reviews—Carson. History of the Medical Department of the University of Pennsylvania, with Sketches of Deceased Professors .................... 362
Circular No. 1, War Department........ 39
Coles on Progressive Locomotor Ataxia,
Correlation of Physical and Vital Forces, 237
Damond. Strictural Lesions of the Skin, Their Pathology and Treatment...... 362
Dalton. Treatise on Physiology and Hygiene for Schools, Families and Colleges............................... 198
Dunlap. Ovariotomy Report of Cases. 158
Elmore. Physician's Hand Book....... 77
Emmett. Accidental and Congenital Atrasia of the Vagina, with a Mode of Operating for Successfully Establishing the Canal....................... 362
Flint. Recherches Experimentales, Sur une Nouvelle Fonction du Foil ....... 117
Hance. The Physician's Medical Compend and Pharmaceutical Formalæ.... 236
Hartshorne. Conspectus of Medical Science, Comprising Manual of Anatomy, Physiology, Chemistry, Materia Medica, Practice of Medicine, Surgery and Obstetrics .......................... 319
Hartshorne. Essentials of the Principles and Practice of Medicine; a Hand Book for Students and Practitioners.. 321
Hebra on Diseases of the Skin ......... 280
Hillier on Diseases of Women.......... 117
Hill. Syphilis and Local Contagious Disorders... .......................... 320
Hibbard. Nature and Time in the Cure of Disease.......................... 403
Hodge. Diseases Peculiar to Women .. 113
Hudson. A Lecture on the Study of Fevers.............................. 280
Judd & White. The Materia Medica in its Scientific Relations............... 237
Klob. Pathological Anatomy of the Female Sexual Organs.................. 281
Lee. Report of Insanity............... 321
Locke. Some thoughts on Education... 320
McElroy on Dinamic Principles and Physiology of Organic Life. How Medicines Produce Their Effect ........... 321
Marshell's Outlines of Physiology, Human and Comparative................ 197
March Schirrhus or Malignant Disease of the Rectum .......................... 319
Medical Formulary...................... 197
Odontalgia, Commonly called Toothache, ................................. 77
Pavy. Treatise on the Function of Digestion, its Disorders and Their Treatment............................... . 443
Pennsylvania Hospital Report.......... 362
Percy. Atropia, its Chemical, Physiological and Therapeutical Action ..... 233
Physician's Visiting List .............. 116
Position in the Treatment of Chloroform Poisoning ........................... 39
Robertson. Manual on Extracting Teeth, 116
Sayre. New Operation for Artificial Anchylosis, Illustrated by two Cases.. 361
Seaton. Hand Book of Vaccination.... 195
Sims on the Microscope in the Diagnosis and Treatment of Sterility...... ..... 363
Smith. Treatise upon the Diseases of Infancy and Childhood ............... 319
Storer. Present Problems in Abdominal Section............................... 237
Storer & Heard on Criminal Abortion, its Nature, its Evidences and its Law.... 157
Theoretical and Practical Treatise on Midwifery................. . ........ 114

Reviews—Thompson. Clinical Lectures upon Diseases of the Urinary Organs. 320
Thomas. Practical Treatise on the Diseases of Women ........ 402
Tilt. Hand Book of Uterine Therapeutics and Diseases of Women ........ 361
Transactions of the Medical Society of the State of North Carolina ........ 156
Transactions of the Indiana State Medical Society ........ 157
Trölsch. Treatise upon Diseases of the Ear, Including the Anatomy of the Organ ........ 360
White's Dental Materia Medica ........ 76
Rights of Medical Experts as Witnesses, 75
Room in the Profession. Letter and Answer ........ 143

### S.

Sarracenia Purpura, as a Remedy for Small Pox ........ 58
Scarlatina, Carbolic Acid in ........ 267
Simpson, Sir Jas. Y., on Medical Progress, 171
Semi-Annual Meeting of the Erie County Medical Society ........ 404
Smith, Eugene, M. D. Case of Capsular Secondary Cataract, with Calcareous Deposit on Anterior Capsule, following Operation by Division ........ 459
Snow, Black ........ 35
Settlement of Controversy between Professors Blackman and Bartholow ........ 119
Solubility of False Diphtheritic Membrane 108
Solubility of Diphtheritic Membrane ........ 180
Spleen, Laceration of in Pregnancy ........ 141
Sponge-Tent, Its Preparation and Use ........ 174
Stomach, Case of Inflammation of ........ 139
Statistics. By H. W. Dean, M. D ........ 206
St. Lawrence County Medical Society ........ 239
Strychnia in Epilepsy ........ 460
Sulphur, Croup Treated by ........ 187
Supra-renal Melasma ........ 468
Surgeon General's Report ........ 280
Surgery, Work on Orthopædic ........ 282
Suture for Vesico-Vaginal Fistula, Inserted by means of a Canula-Needle, with Report of case ........ 396
Sub-Cutaneous Syringe ........ 322
Svapina, or Purified Opium ........ 272
Sweet, Dr. Geo. Jay, M. D., Death of ........ 79
Sweet Quinine and Svapina ........ 240

### T.

Thymic Acid ........ 183
Tooth-Ache, Remedy for ........ 172
Treatment of Ascarides ........ 36
Treatment, "Modern," of Acute Internal Inflammation ........ 146
Treatment of Acute Rheumatism, New Method ........ 175
Treatment of Boils, Whitlows, and Abscesses, by Carbolic Acid ........ 176
Treatment of Malarial Diseases ........ 183
Tuberculosis and Pulmonary Phthisis, A Critical Review ........ 50, 97
Tuum est ........ 477

### U.

Unauthorized Renewal of Prescriptions .. 317
Unbecoming Medical Journalism ........ 481
Use of the Bromides in case of Lead or Mercurial Poisoning ........ 229
Utilization and Contagion ........ 223
Uterine Supporter ........ 482
Uterus, "Inverted," Reduced after four and a half year's duration ........ 364

### V.

Value of Medicine ........ 66
Vaginismus. By Prof. N. S. Davis, M. D. 105
Venereal Disease, Atlas of Dr. Bumstead .. 40
Vesico-Vaginal Fistula. Dr. Emmett ........ 80
Venesection as a means for the Arrest of Unavoidable Hemorrhage in Placenta Previa. By O. C. Gibbs, M. D ........ 310
Vesical Absorption ........ 395

### W.

Whitney, J. O., M. D., Dangers of Opium in Mania a Potu ........ 46
Williams, W. H., M. D., A case of Polypus, 325
Wound of the Abdomen, with Tranverse Division of the Small Intestines in two places, and Division of the Mesenteric Artery ........ 182
Woman's Medical College, New York, Opening of ........ 191
Wounds of the Joint ........ 181

### Books and Pamphlets Received.

The Anatomy and Histology of the Human Eye. By A. Metz. M. D., Professor of Ophthalmology in Charity Hospital Medical College, Cleveland, Ohio. Philadelphia: Office of the Medical and Surgical Reporter.

Dental Materia Medica. Compiled by James W. White. Philadelphia: Published by Samuel S. White, 1868.

Progressive Locomotor Ataxia; being extracts from two papers on "Progressive Locomotor Ataxia, with special reference to its differential diagnosis," read before the Wood County Medical Association, Parkersburg, W. Va., in May and June, 1867. By Walter Coles, M. D.

On Bartholow and Pro's "Liberal Use" of Prize Essays; or prize-essaying made easy, and taught in a single lesson. By Geo. C. Blackman, M. D.

Transactions of the Indiana State Medical Society at its Eighteenth Annual Session, held at Indianapolis, May 19 and 20, 1868.

Announcements from the following Medical Colleges:

The University of Michigan.

Medical Department of Columbia College. Sixty-first Annual Catalogue and Announcement. New York, 1868.

Annual Announcement and Catalogue of the Missouri Medical College, Session of 1868-9.

Twenty-sixth Annual Announcement of Rush Medical College, Chicago, Ill. Session of 1868–9.

Tenth Annual Announcement of the Chicago Medical College, Chicago, Ill. Session of 1868-9.

Announcement of the Fourth Annual Course of Instruction in the St. Louis College of Pharmacy. Session of 1868-9.

Ohio State Medical Society. Annual Address of the retiring President, Edward B. Stevens, M. D , Prof. of Materia Medica in Miami Medical College of Cincinnati.

We have received Part iii of the *Atlas of Venereal Diseases*, by Freeman J. Burnstead, M. D., which is to be completed in five parts. This part fully justifies the opinion so often expressed that when complete it will be the most perfect and valuable work on venereal yet published. The illustrations are the chief attraction, and are sufficient to insure its universal adoption.

